# Development of Species-Specific SCAR Markers, Based on a SCoT Analysis, to Authenticate *Physalis* (Solanaceae) Species

**DOI:** 10.3389/fgene.2018.00192

**Published:** 2018-05-29

**Authors:** Shangguo Feng, Yujia Zhu, Chenliang Yu, Kaili Jiao, Mengying Jiang, Jiangjie Lu, Chenjia Shen, Qicai Ying, Huizhong Wang

**Affiliations:** ^1^Zhejiang Provincial Key Laboratory for Genetic Improvement and Quality Control of Medicinal Plants, College of Life and Environmental Sciences, Hangzhou Normal University, Hangzhou, China; ^2^College of Bioscience and Biotechnology, Hunan Agricultural University, Changsha, China; ^3^The Institute of Vegetable, Zhejiang Academy of Agricultural Sciences, Hangzhou, China

**Keywords:** *Physalis* species, species-specific, SCoT markers, SCAR markers, marker development

## Abstract

*Physalis* is an important genus in the Solanaceae family. It includes many species of significant medicinal value, edible value, and ornamental value. However, many *Physalis* species are easily confused because of their similar morphological traits, which hinder the utilization and protection of *Physalis* resources. Therefore, it is necessary to create fast, sensitive, and reliable methods for the *Physalis* species authentication. Intended for that, in this study, species-specific sequence-characterized amplified region (SCAR) markers were developed for accurate identification of the closely related *Physalis* species *P. angulata*, *P. minima*, *P. pubescens*, and *P. alkekengi* var. *franchetii*, based on a simple and novel marker system, start codon targeted (SCoT) marker. A total of 34 selected SCoT primers yielded 289 reliable SCoT loci, of which 265 were polymorphic. Four species-specific SCoT fragments (SCoT3-1404, SCoT3-1589, SCoT5-550, and SCoT36-520) from *Physalis* species were successfully identified, cloned, and sequenced. Based on these selected specific DNA fragments, four SCAR primers pairs were developed and named ST3KZ, ST3MSJ, ST5SJ, and ST36XSJ. PCR analysis of each of these primer pairs clearly demonstrated a specific amplified band in all samples of the target *Physalis* species, but no amplification was observed in other *Physalis* species. Therefore, the species-specific SCAR primer pairs developed in this study could be used as powerful tools that can rapidly, effectively, and reliably identify and differentiate *Physalis* species.

## Introduction

The genus *Physalis* (Solanaceae family) consists of 75–120 species, which are mainly distributed in American tropical and temperate regions (Chinese Academy of Sciences, 1978; Whitson and Manos, 2005; Wei et al., 2012; Feng et al., 2016b). However, *P*. *angulata*, *P*. *minima*, *P*. *pubescens*, and *P*. *alkekengi* var. *franchetii* are mainly distributed in China (Chinese Academy of Sciences, 1978). They have a variety of pharmacological activities, such as anti-oxidant, anti-inflammatory, and anti-cancer effects, and most of these species have been used as Chinese medicinal herbs for the treatment of malaria, rheumatism, hepatitis, asthma, and cancer for a long time (Ji et al., 2013; Ding et al., 2014; Continuing Professional Education Committee, 2015; Xu et al., 2016). Furthermore, *P*. *alkekengi* var. *franchetii*, a standard *Physalis* medicinal plant, has been included in the Pharmacopoeia of China (Continuing Professional Education Committee, 2015). In addition, some *Physalis* species including *P. philadelphica*, *P. peruviana*, and *P. pubescens* have important ornamental value and edible value, and have been cultivated in many regions of the world, such as China and Europe. In recent years, increasing attention has been paid to the genus *Physalis*, particularly their phytochemical and pharmacology characteristics (Ji et al., 2013; Ding et al., 2014; Li et al., 2014; Yang et al., 2016; Zhang and Tong, 2016; Sun et al., 2017). However, the research on classification and identification of *Physalis* species is weak. The morphological characteristics of *Physalis* species are extremely similar and are easily influenced by their environment and the plant developmental stage, which makes differentiation very difficult and sometimes impossible using morphological methods (Wei et al., 2012; Feng et al., 2016b). In addition, owing to overexploitation and increased urbanization, the natural *Physalis* resources have become endangered, particularly in many areas of China (Feng et al., 2016b). These problems are inhibiting the use of *Physalis* and the development of the *Physalis* industry. Therefore, it is necessary to establish a quick and effective species identification method for the utilization and protection of *Physalis* resources.

In contrast to phenotypic characters, molecular markers are independent of environmental conditions and show higher levels of reliability. They have been widely used as important tools by modern taxonomists to improve phylogenetic analysis and to species authenticate many different plants (Mulpuri et al., 2013; Feng et al., 2015a,b). At present, only a few molecular markers have been applied to the genetic relationship and diversity study of *Physalis* species (Whitson and Manos, 2005; Vargas-Ponce et al., 2011; Wei et al., 2012; Labate and Robertson, 2015; Feng et al., 2016b).

As a simple and novel marker system, start codon targeted (SCoT) marker was developed based on the short conserved region flanking the start codon (ATG) in plant genes (Collard and Mackill, 2009). SCoT marker requires no sequence information and is correlated with functional genes and corresponding traits (Mulpuri et al., 2013). Compared with random amplified polymorphic DNA (RAPDs), inter-simple sequence repeats (ISSR), and simple sequence repeats (SSRs), SCoT employs longer primers (18 nucleotide bases 18-mer) that produce more polymorphisms. It has been extensively used in genetic diversity studies, phylogenetic analysis, and for the marker-assisted breeding of many plants (Collard and Mackill, 2009; Luo et al., 2010, 2011, 2012; Xiong et al., 2011; Guo et al., 2012; Mulpuri et al., 2013; Feng et al., 2015a, 2016a). However, because it is relatively sensitive to experimental conditions and the complexity of PCR products when used as RAPDs and ISSRs, SCoT markers are rarely used directly for species identification. In order to alleviate the problems that arise when using conventional molecular marker techniques, the sequence characterized amplified region (SCAR) marker system was developed (Paran and Michelmore, 1993). As one of the PCR-based genetic markers, SCARs could be derived from RAPD (Joseph et al., 2014), ISSR (Lee et al., 2011), SCoT (Mulpuri et al., 2013), inter-retrotransposon amplified polymorphism (IRAP) (Xiao et al., 2011; Mandoulakani et al., 2015), and intron length polymorphisms (ILP) (Shimada et al., 2009) markers. It represents a specific, defined genomic DNA fragment that is detected by PCR amplification using a pair of specific primers. Compared to conventional molecular markers, SCARs have proven to be simpler, more reproducible and more reliable when used for plant identification at the intra-and/or inter-specific level (Lee et al., 2011; Cirillo et al., 2012; Mulpuri et al., 2013; Marieschi et al., 2016).

The objective of this study was to develop species-specific SCAR markers for identifying four popular *Physalis* species (*P*. *minima*, *P*. *angulata*, *P*. *alkekengi* var. *franchetii*, and *P*. *pubescens*) based on SCoT analysis. Because SCoT is related to functional genes and corresponding traits, SCoT-SCAR has better stability and specificity.

## Materials and Methods

### Plant Materials and DNA Extraction

A total of 20 individuals belonging to four *Physalis* species collected from their natural distribution areas in China were used to screen specific markers (**Table 1**). These individuals are popular medicinal plants in the genus *Physalis*, such as *P*. *alkekengi* var. *franchetii*, which is included in the Chinese Pharmacopoeia (Continuing Professional Education Committee, 2015). Further, 16 individuals of *P*. *angulata*, 10 individuals of *P. pubescens*, 10 individuals of *P*. *alkekengi* var. *franchetii*, and 10 individuals of *P*. *minima* were used to validate the developed SCAR markers (**Supplementary Table S1**). In order to identify and confirm the collected samples at the morphological level, the specimens stored in the herbarium at the Institute of Botany, Chinese Academy of Sciences, China^1^ were used to confirm the collected samples. Genomic DNA was isolated from the fresh, young leaf tissues of all the collected samples and the integrity and quality of the DNA was evaluated as previously described (Feng et al., 2016b).

**Table 1:** The information for all *Physalis* samples used in the SCoT study.

Number	Species Name	Voucher No.	Locality Information	Longitude (E)	Latitude (N)	Altitude
1	*P. minima*	PHZ3001	Qianxi, Tangshan, Hebei, China	118°18′	40°08′	107
2	*P. minima*	PHZ3003	Mudan, Heze, Shandong, China	115°24′	35°15′	53
3	*P. minima*	PHZ3005	Lu’an, Anhui, China	116°31′	31°44′	76
4	*P. minima*	PHZ3004	Lishui, Zhejiang, China	119°55′	28°28′	74
5	*P. angulata*	PHZ0008	Jianggan, Hangzhou, Zhejiang, China	120°12′	30°15′	15
6	*P. angulata*	PHZ0001	Xiaoshan, Hangzhou, Zhejiang, China	120°15′	30°11′	8
7	*P. angulata*	PHZ0005	Luotian, Huanggang, Hubei, China	115°23′	30°47′	76
8	*P. angulata*	PHZ0007	Baohua, Honghe, Yunnan, China	102°20′	23°17′	1871
9	*P. angulata*	PHZ0009	Nanjing University of Chinese Medicine, Najing, Jiangsu, China	118°56′	32°06′	35
10	*P. angulata*	PHZ0010	Linhai, Taizhou, Zhejiang, China	121°08′	28°51′	13
11	*P. angulata*	PHZ0004	Yueqing, Wenzhou, Zhejiang, China	120°58′	28°06′	7
12	*P. angulata*	PHZ0003	Pujiang, Jinhua, Zhejiang, China	121°30′	31°04′	74
13	*P. angulata*	PHZ0006	Xiajin, Dezhou, Shandong, China	116°00′	36°57′	33
14	*P. alkekengi* var. *franchetii*	PHZ4002	Faku, Shenyang, Liaoning, China	123°24′	42°30′	144
15	*P. alkekengi* var. *franchetii*	PHZ4003	Donggang, Dandong, Liaoning, China	124°08′	39°51′	6
16	*P. alkekengi* var. *franchetii*	PHZ4004	Donggang, Dandong, Liaoning, China	124°08′	39°51′	6
17	*P. alkekengi* var. *franchetii*	PHZ4005	Zoucheng, Jinan, Shandong, China	116°59′	35°24′	85
18	*P. pubescens*	PHZ2001	Faku, Shenyang, Liaoning, China	123°24′	42°30′	144
19	*P. pubescens*	PHZ2004	Chaoyang, Zhaodong, Heilongjiang, China	126°15′	45°52′	129
20	*P. pubescens*	PHZ2007	Nong’an, Changchun, Jilin, China	125°10′	44°25′	196

### SCoT-PCR

Following a study by Collard and Mackill (2009), a total of 36 SCoT primers, synthesized by the Shanghai Sangon Biological Engineering Technology and Service Co., Ltd., China, were used for an initial primer screen, which utilized two samples of each tested species. These primers produced clearly separated bands and allowed stable and rich polymorphisms to be selected (**Table 2**). The PCR reactions were conducted in a total volume of 20 μl containing 1 × PCR buffer [200 mM Tris–HCl (pH 8.8), 100 mM KCl, 100 mM (NH_4_)_2_SO_4_, 20 mM MgCl_2,_ 1% TritonX-100], 0.4 mM dNTPs, 0.5 μM each primer, 1 U *Taq* DNA polymerase (Beijing Dingguo Changsheng Biotechnology Co., Ltd., China), and genomic DNA template 50 ng. The amplification was performed using the following PCR program: 5 min at 94°C, followed by 35 cycles of 50 s at 94°C, 50 s at 50–60°C (depending on the annealing temperature of each primer), 1.5 min at 72°C, and a final extension at 72°C for 10 min. The PCR was performed in a gradient thermal cycler (A200) (Hangzhou Longgene Scientific Instruments Co., Ltd., Zhejiang, China). The PCR products were separated on 1.5% (W/V) agarose gel using Trans2K DNA markers (TransGen Biotech, Beijing, China) as size standards in Tris-acetate buffer stained with GelStain (TransGen Biotech) and photographed under UV light. The experiments were repeated at least two times.

**Table 2:** Genetic polymorphism of 34 SCoT primers.

Primer code	Primer Sequence (5′-3′)	Annealing temperature (°C)	No. of amplified loci	No. of Polymorphic loci	Polymorphic loci (%)
1	CAACAATGGCTACCACCA	49.86	7	7	100.0
2	CAACAATGGCTACCACCC	50.73	6	4	66.7
3	CAACAATGGCTACCACCG	51.27	9	9	100.0
4	CAACAATGGCTACCACCT	49.5	10	8	80.0
5	CAACAATGGCTACCACGA	50.1	7	5	71.4
6	CAACAATGGCTACCACGC	52.05	11	11	100.0
7	CAACAATGGCTACCACGG	51.27	11	11	100.0
8	CAACAATGGCTACCACGT	50.41	12	12	100.0
9	CAACAATGGCTACCAGCA	50.32	4	3	75.0
10	CAACAATGGCTACCAGCC	51.19	6	5	83.3
11	AAGCAATGGCTACCACCA	51.37	6	5	83.3
12	ACGACATGGCGACCAACG	55.93	7	7	100.0
13	ACGACATGGCGACCATCG	55.39	8	8	100.0
14	ACGACATGGCGACCACGC	58.58	12	11	91.7
15	ACGACATGGCGACCGCGA	59.85	9	8	88.9
16	ACCATGGCTACCACCGAC	54.05	13	13	100.0
17	ACCATGGCTACCACCGAG	53.71	12	12	100.0
18	ACCATGGCTACCACCGCC	57.09	4	3	75.0
19	ACCATGGCTACCACCGGC	57.09	7	6	85.7
20	ACCATGGCTACCACCGCG	57.53	6	4	66.7
21	ACGACATGGCGACCCACA	56.65	10	9	90.0
22	AACCATGGCTACCACCAC	51.85	13	13	100.0
23	CACCATGGCTACCACCAG	52.43	11	10	90.9
24	CACCATGGCTACCACCAT	51.58	7	5	71.4
25	ACCATGGCTACCACCGGG	56.35	6	6	100.0
26	ACCATGGCTACCACCGTC	54.05	5	5	100.0
27	ACCATGGCTACCACCGTG	54.37	9	9	100.0
29	CCATGGCTACCACCGGCC	57.9	10	10	100.0
30	CCATGGCTACCACCGGCG	58.32	5	5	100.0
32	CCATGGCTACCACCGCAC	55.94	12	12	100.0
33	CCATGGCTACCACCGCAG	55.62	9	5	55.6
34	ACCATGGCTACCACCGCA	56.27	6	6	100.0
35	CATGGCTACCACCGGCCC	57.9	7	7	100.0
36	GCAACAATGGCTACCACC	51.53	12	11	91.7
Average	–	–	8.5	7.8	90.2
Total	–	–	289	265	

### Analysis of SCoT Profiles

The SCoT amplified bands were scored visually, but this was assisted by Quantity One software (Version 4.6.2, Bio-Rad Technical Service Department, United States). Only clear, unambiguous, and reproducible SCoT fragments were scored as present (1) or absent (0). The NTSYS-pc version 2.10e software was used to conduct the cluster analysis (Rohlf, 2000). The unweighted pair group method with arithmetic mean (UPGMA) was used to construct a dendrogram based on similarity matrices that had been calculated along with a simple matching (SM) coefficient (Nei and Li, 1979). In addition, a UPGMA analysis was performed based on the genetic distances using MEGA 6.0 software.

### Selection, Cloning, and Sequencing of Specific SCoT Fragments

The SCoT profiles were compared to select amplicons present in a particular species and their absence in all the other species, which meant that they could be considered as species-specific markers. Several suitable marker bands were obtained using primers SCoT2, SCoT3, SCoT4, SCoT5, SCoT19, SCoT22, and SCoT36 (**Table 3**). The selected marker bands were excised from 1.5% agarose gel and were purified using a DNA Fragment Quick Purification/Recovery Kit (Beijing Dingguo Changsheng Biotechnology Co., Ltd.). The purified DNA fragments were cloned in a pUCm-T vector (Shanghai Sunny Biotechnology Co., Ltd.) and incorporated into ultra-competent *Escherichia coli* strain Trans5α cells. The transformed bacterial colonies were selected by colony PCR and clones with correctly sized inserts were sequenced using M13 forward and M13 reverse primers by the Shanghai Sunny Biotechnology Co., Ltd.

**Table 3:** Species-specific SCoT loci for *Physalis* species.

Primer name	Species-specific locus	Sequence length (bp)	*Physalis* species	GenBank accession number
SCoT2	SCoT2-730	730	*P. alkekengi* var. *franchetii*	–
SCoT3	SCoT3-1404	1404	*P. angulata*	MF566104
SCoT3	SCoT3-1589	1589	*P. pubescens*	MF566105
SCoT4	SCoT4-1412	1412	*P. minima*	–
SCoT5	SCoT5-550	550	*P. alkekengi* var. *franchetii*	MF566106
SCoT19	SCoT19-590	590	*P. angulata*	–
SCoT22	SCoT22-662	662	*P. alkekengi* var. *franchetii*	–
SCoT22	SCoT22-537	537	*P. pubescens*	–
SCoT36	SCoT36-520	520	*P. minima*	MF566107
SCoT36	SCoT36-462	462	*P. angulata*	–
SCoT36	SCoT36-417	417	*P. pubescens*	–

### Sequence Data Analysis, and SCAR Primers Design and Validation

The sequence similarities of the obtained sequences were identified using the nucleotide databases of BLASTN^2^ program and the sequences were deposited in GenBank (GenBank accession numbers: MF566104, MF566105, MF566106, and MF566107) (Clark et al., 2016) (**Table 4**). The SCAR primers were designed using Primer Premier 5 software (Lalitha, 2000). Four SCAR primer pairs, named ST3KZ, ST3MSJ, ST5SJ, and ST36XSJ, were designed based on the sequenced SCoT fragments (**Table 4**). The designed primers were 19- to 22-mers with annealing temperatures (*T_m_*) between 49.9 and 61.9°C. They were based on the obtained specific SCoT fragments and synthesized by Sangon Biotech (Shanghai) Co., Ltd. SCAR amplification was performed in 20 μl of reaction mixture containing 1 × PCR buffer [200 mM Tris–HCl (pH 8.8), 100 mM KCl, 100 mM (NH_4_)_2_SO_4_, 20 mM MgCl_2,_ 1% TritonX-100], 0.4 mM of dNTPs, 0.5 μM of forward primer, 0.5 μM of reverse primer, 50 ng of DNA, and 1 U *Taq* DNA polymerase (Beijing Dingguo Changsheng Biotechnology Co., Ltd.). The SCAR-PCR amplifications were carried out with an initial denaturation at 94°C for 5 min, followed by 35 cycles at 94°C for 50 s, a working annealing temperature, which depended on each SCAR primer pair (**Table 4**), for 50 s, 72°C for 1.5 min, and with a final extension at 72°C for 10 min. The amplification products were resolved by electrophoresis on 1.5% agarose gel and detected by GelStain (TransGen Biotech) staining.

**Table 4:** Characteristics of SCAR primer pairs developed from polymorphic SCoT primers.

SCoT primer	SCAR primer	SCAR primer sequence (5′-3′)	*T_m_* (°C)	Working annealing temperature (°C)	Aplicon length (bp)	Target species
SCoT3	ST3KZ	For GCACCTCGCAATACCGCACA	61.9	65	1102	*P. angulata*
		Rev TCCCCAACTCGAATCAACCG	59.8			
SCoT3	ST3MSJ	For ATTGATAGGGTAAACCATG	51.1	56	1479	*P. pubescens*
		Rev CTGTAATAAAACAAAAGCG	49.9			
SCoT5	ST5SJ	For AGGGTGTGGGGCTTCTTTA	57.6	61	463	*P. alkekengi* var. *franchetii*
		Rev GGCTGCTCTGGCCTCTGTA	61.9			
SCoT36	ST36XSJ	For TTGCAAACTATCTTGTGAGTCG	53.7	60	444	*P. minima*
		Rev CCACCAGAATGTACTTGTTACG	54.7			

## Results

### SCoT Analysis

**FIGURE 1:**
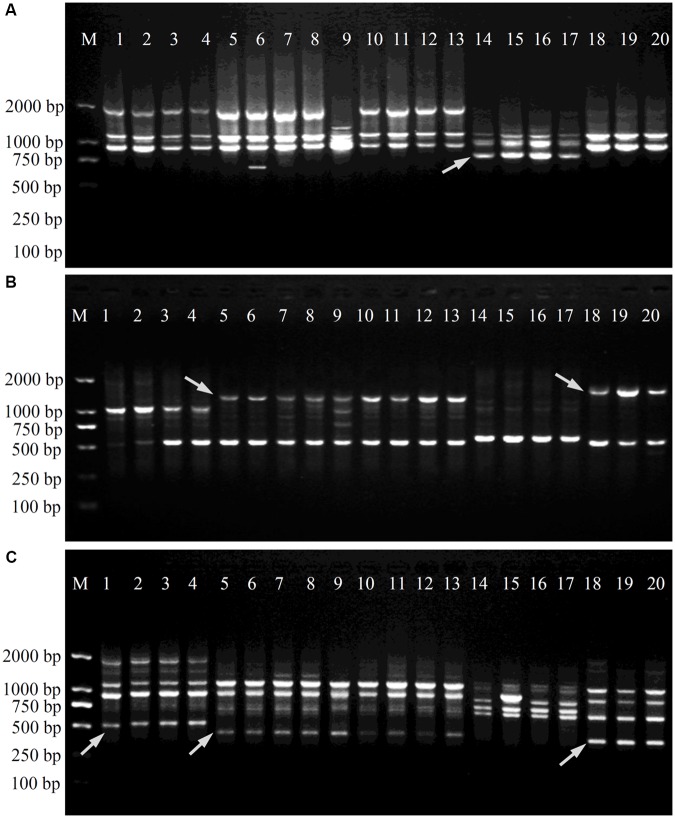
Amplification profiles of primers SCoT2 **(A)**, SCoT3 **(B)** and SCoT36 **(C)**. Lane M: Trans2K DNA Marker; Lanes 1–4: four individuals belonging to *P. minima*, Lanes 5–13: nine individuals belonging to *P. angulata*, Lanes 14–17: four individuals belonging to *P. alkekengi* var. *franchetii*, Lanes 18–20: three individuals belonging to *P. pubescens*. Details of *Physalis* individuals given in **Table 1**; *Arrowheads* represent specific amplified bands in all individuals of target *Physalis* species.

After the initial primer screening, 34 SCoT primers that produced clear and repeatable polymorphic patterns were chosen for further study (**Table 2**). These 34 primers produced 289 reliable SCoT bands, of which 265 bands were polymorphic. The polymorphic bands per primer ranged from 3 (SCoT9 and SCoT18) to 13 (SCoT22), with an average of 7.8. The percentage of polymorphic bands in each primer varied from 55.6 to 100.0%, with an average of 90.2%. Three representative profiles (SCoT2, SCoT3, and SCoT36) are shown in **Figure 1**.

**FIGURE 2:**
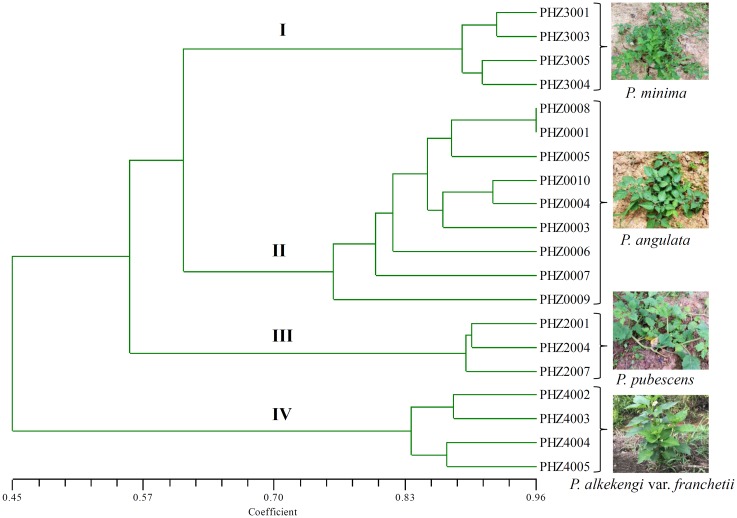
Dendrogram of *Physalis* species in this study using UPGMA cluster analysis based on genetic similarities of DNA fingerprinting patterns from the 34 SCoT primers. Numbers (I–IV) indicates *Physalis* samples were grouped into four groups with a similarity of 0.622.

A total of 289 loci were accounted for the phylogenetic analysis. The genetic similarity among *Physalis* samples was estimated using the binary data matrices produced by SCoTs (**Supplementary Table S2**). The UPGMA dendrogram grouped *Physalis* samples into four main groups with a similarity of 0.622 (**Figure 2**). The UPGMA tree showed that all of the samples from the same species were grouped into one group and that an obvious boundary existed between species (group I contained the four samples from *P. minima*, group II contained the nine samples from *P. angulata*, group III contained the three samples from *P. pubescens*, while all the samples from *P. alkekengi* var. *franchetii*, which were far apart from any of the other *Physalis* species, were grouped into IV) (**Figure 2**). The similar results were obtained by UPGMA analysis based on the genetic distances using MEGA 6.0 software (**Supplementary Figure S1**).

### Identification, Sequence Analysis, and Development of the SCAR Markers

A total of 11 SCoT fragments, each specific for a particular species and absent in the SCoT profiles of all the other species samples (SCoT2-730, SCoT5-550, and SCoT22-662 were specific for *P. alkekengi* var. *franchetii*; SCoT3-1404, SCoT19-590, and SCoT36-462 were specific for *P. angulata*; SCoT3-1589, SCoT22-537, and SCoT36-417 were specific for *P. pubescens*; and SCoT4-1412 and SCoT36-520 were specific for *P. minima*), were identified (**Table 3**). During cloning and sequencing, 7 of the 11 specific SCoT fragments were rejected due to technical problems (these fragments were not successfully cloned, leading to the failure of sequencing). The remaining four fragments, SCoT3-1404, SCoT3-1589, SCoT5-550, and SCoT36-520, were successfully cloned and sequenced. Their sequences were deposited in GenBank and their accession numbers are shown in **Table 3**. The blast results showed that most of the SCoT fragments had some similarities with other sequences deposited in GenBank. SCoT3-1404 had 80% identity with the *Solanum lycopersicum* chromosome ch10, a complete genome (GenBank accession: HG975522). SCoT3-1589 showed 80% identity with the RNA-directed DNA polymerase homolog mRNA of *Daucus carota* subsp. *sativus* (GenBank accession: XM_017389806), and SCoT36-520 had a high similarity (91%) with the corresponding region of mRNA from *Brassica oleracea* var. *oleracea* (GenBank accession: XM_013769290). However, SCoT5-550 showed no similarity with other known sequences at the sequence-similarity level.

### SCAR Markers Specific for *Physalis* Species

**FIGURE 3:**
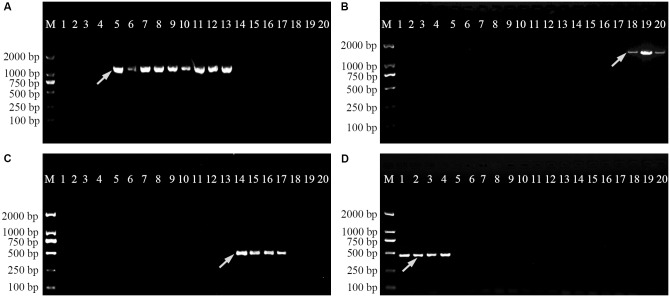
Amplification profiles of primer pairs ST3KZ **(A)**, ST3MSJ **(B)**, ST5SJ **(C)**, and ST36XSJ **(D)** in 20 *Physalis* samples. Lane M: Trans2K DNA Marker; Lanes 1–4: four individuals belonging to *P. minima*, Lanes 5–13: nine individuals belonging to *P. angulata*, Lanes 14–17: four individuals belonging to *P. alkekengi* var. *franchetii*, Lanes 18–20: three individuals belonging to *P. pubescens*. Details of *Physalis* individuals given in **Table 1**; *Arrowheads* represent species-specific markers.

**FIGURE 4:**
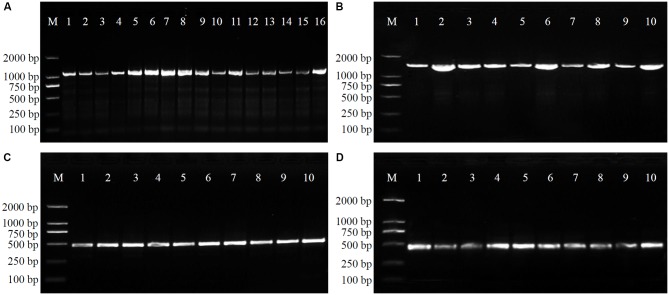
Amplification profiles of primer pairs ST3KZ in 16 *P. angulata* individuals **(A)**, ST3MSJ in 10 *P. pubescens* individuals **(B)**, ST5SJ in 10 *P. alkekengi* var. *franchetii* individuals **(C)**, and ST36XSJ in 10 *P. minima* individuals **(D)**. Details of *Physalis* individuals given in **Supplementary Table S1**.

In order to verify the specificity of the SCAR markers, each designed primer pair was tested after amplification using DNA extracted from 20 samples of four *Physalis* species (**Table 1**). After optimization of the PCR conditions, the optimum working annealing temperatures of these primer pairs (ST3KZ, ST3MSJ, ST5SJ, and ST36XSJ) were 65°C, 56°C, 61°C, and 60°C, respectively (**Table 4**). The amplification profiles of the SCAR primers are shown in **Figure 3**. The profile of the primer pair ST3KZF/R showed (**Figure 3A**) that a clear specific band representing 1102 bp was detected in all *P. angulata* samples, but no PCR band was found in the samples from *P. minima*, *P. pubescens*, and *P. alkekengi* var. *franchetii*. Similarly, all the tested individuals from the remaining three species contained a specific band detected by ST3MSJF/R (a band with 1479 bp for *P. pubescens*, **Figure 3B**), ST5SJF/R (a band with 463 bp for *P. alkekengi* var. *franchetii*, **Figure 3C**), and ST36XSJF/R (a band with 444 bp for *P. minima*, **Figure 3D**). The SCAR markers were further validated in 16 *P*. *angulata*, 10 *P. pubescens*, 10 *P*. *alkekengi* var. *franchetii*, and 10 *P*. *minima* individuals and the amplification of the markers at 1102, 1479, 463, and 444 bp ascertained the parentage of the target species (**Figure 4**).

## Discussion

In recent years, *Physalis* species have attracted the attention of many different scientists because of their significant nutritional value, edible fruit, and potential medicinal value. Many *Physalis* species have been used as raw materials to extract the active chemical constituents for new drug development (Fang et al., 2012; Ji et al., 2013; Ding et al., 2014; Li et al., 2014; Sang-Ngern et al., 2016; Zhang and Tong, 2016). Traditionally, *Physalis* identification was mainly dependent on morphological traits (Axelius, 1996; Martinez, 1998). However, the morphological features between *Physalis* species are extremely similar. In addition, morphological characteristics are often influenced by plant variation and growth habitats (Whitson and Manos, 2005). These problems have greatly hindered the utilization and protection of *Physalis* resources. DNA molecular marker technologies provide fast, effective, and accurate methods for identifying similar looking plants.

Several different molecular markers, including ISSR, SSR, InDel, SNP, ITS2, and *psbA-trnH* intergenic region, have been used for evaluating the genetic diversity and relationships of *Physalis* species (Vargas-Ponce et al., 2011; Wei et al., 2012; Garzon-Martinez et al., 2015; Zamora-Tavares et al., 2015; Feng et al., 2016b, 2018). This is the first study to identify *Physalis* species based on SCoT analysis. SCoT is a novel, simple and reliable gene-targeted marker technique, and has commonly been used to reveal genetic diversity and relationships in a wide range of plants, such as mango (Luo et al., 2010), grape (Guo et al., 2012), *Mangifera indica* (Luo et al., 2012), *Dendrobium* (Feng et al., 2015a), and *Chrysanthemum morifolium* (Feng et al., 2016a).

In this study, the amplified SCoT loci contained 265 polymorphic loci, which represented a polymorphism rate of 90.2%. This was higher than the polymorphic loci rate detected among mango cultivars (65.82%) (Luo et al., 2011), and *M. indica* (73.82%) (Luo et al., 2012), but similar to the proportion reported among *C. morifolium*, which was about 90% (Feng et al., 2016a). The high polymorphic rates detected in this study indicated that the SCoT technique has the potential ability to differentiate *Physalis* species. In recent years, the taxonomy of *Physalis* is regarded as one of the most intricate challenges in Solanaceae (Whitson and Manos, 2005; Olmstead et al., 2008; Feng et al., 2016b). In our previous studies, we found that several previously defined infrageneric taxa of *Physalis* are not monophyletic, and suggested that *P. alkekengi* var. *franchetii* should be recognized as a small genus (Feng et al., 2016b, 2018). In Whitson and Manos’ (2005) study, it was indicated that *P. alkekengi*, *P. carpenter*, and *P. microphysa* were atypical *Physalis* species and should be recognized as three small genera. In this study, the dendrogram constructed using the UPGMA method contained four groups, and indicated that different samples from the same *Physalis* species can be grouped on one branch (**Figure 2**). All samples from *P. alkekengi* var. *franchetii* constituted a separate group (IV), which was distant from any other *Physalis* species and this result confirmed with the findings of previous studies (Whitson and Manos, 2005; Feng et al., 2016b).

The SCoT markers could be used for species authentication by converting the species-specific SCAR markers for some other plant species. For example, Mulpuri et al. (2013) developed a SCAR marker based on SCoT analysis, which could be used to identify the toxic and non-toxic accessions of *Jatropha curcas*. A SCoT-derived SCAR marker was developed to distinguish tall/dwarf trait in arecanut (Rajesh et al., 2016). Hao et al. (2018) developed a SCoT-Based SCAR Marker for rapid authentication of *Taxus Media* among other related *Taxus* species (*T. chinensis*, *T. cuspidate*, and *T. fauna*) (Hao et al., 2018). In this study, SCoT markers generated 11 species-specific SCoT bands, four (SCoT3-1404, SCoT3-1589, SCoT5-550, and SCoT36-520) of which were successfully cloned, sequenced, and converted into SCAR markers. Usually, SCAR markers have been developed from traditional fingerprinting methods, such as RAPD (Joseph et al., 2014), ISSR (Lee et al., 2011), and AFLP amplicons (Choi et al., 2008).

Each of the SCAR markers (ST3KZ, ST3MSJ, ST5SJ, and ST36XSJ) developed in the study produced a specific amplicon of a certain length for each target *Physalis* species, but no amplicons were produced by other, non-target *Physalis* species (**Figures 3**, **4** and **Table 4**). These results revealed that SCAR markers might be used to identify and differentiate *Physalis* species. Of course, in the future, we will collect more *Physalis* species to further verify the reliability of our results. An increasing number of studies have reported that SCAR markers have been used to authenticate a variety of plant species, such as *Panax japonicas* (Choi et al., 2008), *Casuarina equisetifolia* (Ghosh et al., 2011), *Jatropha curcas* (Mulpuri et al., 2013), *Rosa* (Bashir et al., 2014), *Commiphora* (Sairkar et al., 2016), and *Dasypyrum* species (Hu et al., 2017).

Of course, in order to identify and classify *Physalis* species more accurately, it is best to conduct relevant studies from the genome level. At present, the complete chloroplast genome of *P. peruviana* have been sequenced (GenBank accession: NC_026570), which provides a new method for more accurate identification and phylogenetic reconstruction of *Physalis* species in the future.

## Conclusion

A fast, sensitive, and reliable method for identifying *Physalis* species based on SCAR markers was developed from SCoT fragments that were specific for four target *Physalis* species. The specific primer pairs developed in this study clearly demonstrated that the specific amplicon was only present in the target *Physalis* species, while no amplification was observed in the other *Physalis* non-target species. The resulting specific SCAR primers were shown to be powerful tools that could be used to rapidly and reliably screen *Physalis* species.

## Author Contributions

SF and HW conceived and designed the study. HW, SF, and JL collected the plant samples. CY, KJ, YZ, MJ, and QY performed the experiments. SF, KJ, JL, and CS analyzed the data. SF and HW wrote the manuscript.

## Conflict of Interest Statement

The authors declare that the research was conducted in the absence of any commercial or financial relationships that could be construed as a potential conflict of interest.

## Footnotes

1 http://www.cvh.ac.cn/

2 https://blast.ncbi.nlm.nih.gov/Blast.cgi

## Supplementary Material

FIGURE S1 A UPGMA analysis for tested *Physalis* species in this study was performed based on the genetic distances using MEGA 6.0 software. Numbers (I–IV) indicates *Physalis* samples were grouped into four groups.

TABLE S1 List of *Physalis* samples used for SCAR markers validation.

TABLE S2 The pairwise similarity coefficient calculated by SCoT markers.
