# Supplementary figures and images for: Development of Species-Specific SCAR Markers, Based on a SCoT Analysis, to Authenticate Physalis (Solanaceae) Species
Source: Front Genet. 2018 May 29;9:192. doi: 10.3389/fgene.2018.00192 (PMC5992434; doi:10.3389/fgene.2018.00192)

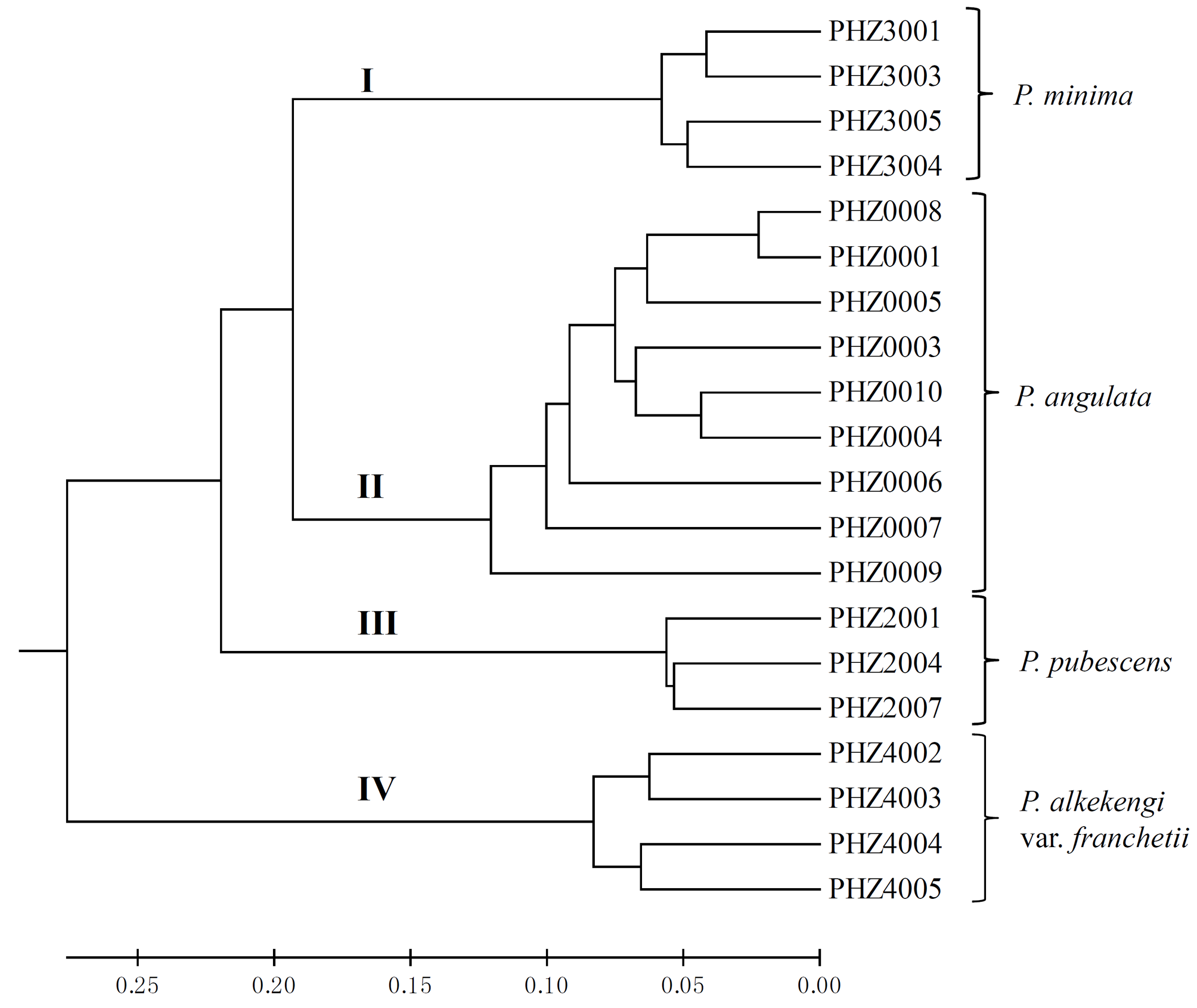

Supplement: FIGURE S1 — A UPGMA analysis for tested Physalis species in this study was performed based on the genetic distances using MEGA 6.0 software. Numbers (I–IV) indicates Physalis samples were grouped into four groups. [file Image_1.TIF]
