# Supplementary material for: Development of Species-Specific SCAR Markers, Based on a SCoT Analysis, to Authenticate Physalis (Solanaceae) Species
Source: Front Genet. 2018 May 29;9:192. doi: 10.3389/fgene.2018.00192 (PMC5992434; doi:10.3389/fgene.2018.00192)
Supplement: TABLE S1 — List of Physalis samples used for SCAR markers validation. [file Table_1.DOC]

Supplementary Table 1 List of *Physalis* samples used for SCAR markers validation

| Species Name | Number | Voucher No. | Locality information | Longitude (E) | Latitude (N) | Altitude |
| --- | --- | --- | --- | --- | --- | --- |
| *P. minima* | 1 | PHZ3001 | Qianxi, Tangshan, Hebei, China | 118°18′ | 40°08′ | 107 |
|  | 2 | PHZ3003 | Mudan, Heze, Shandong, China | 115°24′ | 35°15′ | 53 |
|  | 3 | PHZ3005 | Lou’An, Anhui, China | 116°31′ | 31°44′ | 76 |
|  | 4 | PHZ3004 | Lishui, Zhejiang, China | 119°55′ | 28°28′ | 74 |
|  | 5 | PHZ3002 | Pingdingshan, Henan, China | 113°11′ | 33°46′ | 124 |
|  | 6 | PHZ3006 | Dongchangfu, Liaocheng, Shandong, China | 115°58′ | 36°26′ | 33 |
|  | 7 | PHZ3007 | Dongming, Hezhe, Shandong, China | 115°05′ | 35°17′ | 60 |
|  | 8 | PHZ3008 | Weifang, Shandong, China | 119°09′ | 36°42′ | 31 |
|  | 9 | PHZ3009 | Yunlong, Xuzhou, Jiangsu, China | 117°16′ | 34°12′ | 32 |
|  | 10 | PHZ3010 | Gulou, Fuzhou, Fujian, China | 119°17′ | 26°04′ | 31 |
| *P. angulata* | 1 | PHZ0008 | Jianggan, Hangzhou, Zhejiang, China | 120°12′ | 30°15′ | 15 |
|  | 2 | PHZ0001 | Xiaoshan, Hangzhou, Zhejiang, China | 120°15′ | 30°11′ | 8 |
|  | 3 | PHZ0005 | Luotian, Huanggang, Hubei, China | 115°23′ | 30°47′ | 76 |
|  | 4 | PHZ0007 | Baohua, Honghe, Yunnan, China | 102°20′ | 23°17′ | 1871 |
|  | 5 | PHZ0009 | Nanjing University of Chinese Medicine, Najing, Jiangsu, China | 118°56′ | 32°06′ | 35 |
|  | 6 | PHZ0010 | Linhai, Taizhou, Zhejiang, China | 121°08′ | 28°51′ | 13 |
|  | 7 | PHZ0004 | Yueqing, Wenzhou, Zhejiang, China | 120°58′ | 28°06′ | 7 |
|  | 8 | PHZ0003 | Pujiang, Jinhua, Zhejiang, China | 121°30′ | 31°04′ | 74 |
|  | 9 | PHZ0006 | Xiajin, Dezhou, Shandong, China | 116°00′ | 36°57′ | 33 |
|  | 10 | PHZ0002 | Lin’an, Hangzhou, Zhejaing, China | 119°43′ | 30°14′ | 39 |
|  | 11 | PHZ0011 | Xuanwu, Nanjing, Jangsu, China | 118°47′ | 32°03′ | 12 |
|  | 12 | PHZ0012 | Deqing, Huzhou, Zhejiang, China | 120°02′ | 30°32′ | 8 |
|  | 13 | PHZ0013 | Yiwu, Jinhua, Zhejiang, China | 120°04′ | 29°18′ | 73 |
|  | 14 | PHZ0014 | Ninghai, Ningbo, Zhejiang, China | 121°25′ | 29°17′ | 35 |
|  | 15 | PHZ0015 | Jiaojiang, Taizhou, Zhejiang, China | 121°26′ | 28°40′ | 9 |
|  | 16 | PHZ0016 | Xuyang, Jiujiang, Jiangxi, China | 115°59′ | 29°43′ | 87 |
| *P. alkekengi* var. *franchetii* | 1 | PHZ4002 | Faku, Shenyang, Liaoning, China | 123°24′ | 42°30′ | 144 |
|  | 2 | PHZ4003 | Donggang, Dandong, Liaoning, China | 124°08′ | 39°51′ | 6 |
|  | 3 | PHZ4004 | Donggang, Dandong, Liaoning, China | 124°08′ | 39°51′ | 6 |
|  | 4 | PHZ4005 | Zoucheng, Jinan, Shandong, China | 116°59′ | 35°24′ | 85 |
|  | 5 | PHZ4001 | Nong’an, Changchun, Jilin, China | 125°10′ | 44°25′ | 196 |
|  | 6 | PHZ4006 | Zoucheng, Jinan, Shandong, China | 117°02′ | 36°41′ | 75 |
|  | 7 | PHZ4007 | Qing’an, suihua, Heilongjiang, China | 127°30′ | 46°52′ | 190 |
|  | 8 | PHZ4008 | Nong’an, Changchun, Jilin, China | 125°10′ | 44°25′ | 196 |
|  | 9 | PHZ4009 | Chaoyang, Changchun, Jilin, China | 125°16′ | 43°49′ | 230 |
|  | 10 | PHZ4010 | Baiquan, Qiqiha’er, Heilongjiang, China | 126°05′ | 47°35′ | 232 |
| *P. pubescens* | 1 | PHZ2001 | Faku, Shenyang, Liaoning, China | 123°24′ | 42°30′ | 144 |
|  | 2 | PHZ2004 | Chaoyang, Zhaodong, Heilongjiang, China | 126°15′ | 45°52′ | 129 |
|  | 3 | PHZ2007 | Nong’an, Changchun, Jilin, China | 125°10′ | 44°25′ | 196 |
|  | 4 | PHZ2002 | Guta, Jinzhou, Liaoning, China | 121°07′ | 41°06′ | 27 |
|  | 5 | PHZ2003 | Changhai, Dalian, Liaoning, China | 122°35′ | 39°16′ | 123 |
|  | 6 | PHZ2005 | Baiquan, Qiqiha’er, Heilongjiang, China | 126°05′ | 47°35′ | 232 |
|  | 7 | PHZ2006 | Aihui, Heihe, Heilongjiang, China | 127°31′ | 50°14′ | 120 |
|  | 8 | PHZ2008 | Nong’an, Changchun, Jilin, China | 125°10′ | 44°25′ | 196 |
|  | 9 | PHZ2009 | Dongchang, Tonghua, Jilin, China | 125°56′ | 41°43′ | 374 |
|  | 10 | PHZ2010 | Mudanjiang, Heilongjiang, China | 129°37′ | 44°33′ | 234 |
