# Supplementary material for: Development of Species-Specific SCAR Markers, Based on a SCoT Analysis, to Authenticate Physalis (Solanaceae) Species
Source: Front Genet. 2018 May 29;9:192. doi: 10.3389/fgene.2018.00192 (PMC5992434; doi:10.3389/fgene.2018.00192)
Supplement: TABLE S2 — The pairwise similarity coefficient calculated by SCoT markers. [file Table_2.DOC]

Supplementary Table 2 The pairwise similarity coefficient calculated by SCoT markers

|  | 1 | 2 | 3 | 4 | 5 | 6 | 7 | 8 | 9 | 10 | 11 | 12 | 13 | 14 | 15 | 16 | 17 | 18 | 19 | 20 |
| --- | --- | --- | --- | --- | --- | --- | --- | --- | --- | --- | --- | --- | --- | --- | --- | --- | --- | --- | --- | --- |
| 1 | 1.000 |  |  |  |  |  |  |  |  |  |  |  |  |  |  |  |  |  |  |  |
| 2 | 0.917 | 1.000 |  |  |  |  |  |  |  |  |  |  |  |  |  |  |  |  |  |  |
| 3 | 0.893 | 0.900 | 1.000 |  |  |  |  |  |  |  |  |  |  |  |  |  |  |  |  |  |
| 4 | 0.858 | 0.886 | 0.903 | 1.000 |  |  |  |  |  |  |  |  |  |  |  |  |  |  |  |  |
| 5 | 0.602 | 0.623 | 0.606 | 0.626 | 1.000 |  |  |  |  |  |  |  |  |  |  |  |  |  |  |  |
| 6 | 0.612 | 0.633 | 0.609 | 0.637 | 0.955 | 1.000 |  |  |  |  |  |  |  |  |  |  |  |  |  |  |
| 7 | 0.609 | 0.616 | 0.606 | 0.633 | 0.875 | 0.872 | 1.000 |  |  |  |  |  |  |  |  |  |  |  |  |  |
| 8 | 0.574 | 0.609 | 0.571 | 0.592 | 0.806 | 0.817 | 0.813 | 1.000 |  |  |  |  |  |  |  |  |  |  |  |  |
| 9 | 0.606 | 0.619 | 0.609 | 0.606 | 0.772 | 0.782 | 0.785 | 0.744 | 1.000 |  |  |  |  |  |  |  |  |  |  |  |
| 10 | 0.637 | 0.644 | 0.619 | 0.647 | 0.869 | 0.865 | 0.827 | 0.827 | 0.792 | 1.000 |  |  |  |  |  |  |  |  |  |  |
| 11 | 0.640 | 0.647 | 0.623 | 0.637 | 0.879 | 0.875 | 0.810 | 0.810 | 0.772 | 0.913 | 1.000 |  |  |  |  |  |  |  |  |  |
| 12 | 0.588 | 0.588 | 0.581 | 0.588 | 0.855 | 0.858 | 0.813 | 0.785 | 0.740 | 0.851 | 0.879 | 1.000 |  |  |  |  |  |  |  |  |
| 13 | 0.630 | 0.623 | 0.592 | 0.616 | 0.820 | 0.830 | 0.768 | 0.740 | 0.685 | 0.820 | 0.830 | 0.830 | 1.000 |  |  |  |  |  |  |  |
| 14 | 0.516 | 0.502 | 0.491 | 0.450 | 0.408 | 0.405 | 0.464 | 0.433 | 0.481 | 0.429 | 0.412 | 0.419 | 0.460 | 1.000 |  |  |  |  |  |  |
| 15 | 0.498 | 0.471 | 0.460 | 0.443 | 0.408 | 0.401 | 0.429 | 0.394 | 0.464 | 0.401 | 0.412 | 0.401 | 0.443 | 0.875 | 1.000 |  |  |  |  |  |
| 16 | 0.505 | 0.491 | 0.467 | 0.433 | 0.426 | 0.412 | 0.436 | 0.398 | 0.460 | 0.415 | 0.412 | 0.381 | 0.419 | 0.827 | 0.855 | 1.000 |  |  |  |  |
| 17 | 0.491 | 0.478 | 0.481 | 0.446 | 0.429 | 0.412 | 0.446 | 0.401 | 0.457 | 0.419 | 0.412 | 0.388 | 0.415 | 0.824 | 0.830 | 0.869 | 1.000 |  |  |  |
| 18 | 0.529 | 0.529 | 0.533 | 0.574 | 0.581 | 0.599 | 0.567 | 0.543 | 0.588 | 0.588 | 0.564 | 0.550 | 0.612 | 0.502 | 0.488 | 0.484 | 0.484 | 1.000 |  |  |
| 19 | 0.561 | 0.561 | 0.578 | 0.578 | 0.557 | 0.574 | 0.571 | 0.540 | 0.588 | 0.571 | 0.547 | 0.533 | 0.578 | 0.519 | 0.474 | 0.484 | 0.505 | 0.893 | 1.000 |  |
| 20 | 0.543 | 0.529 | 0.540 | 0.540 | 0.574 | 0.578 | 0.554 | 0.529 | 0.578 | 0.554 | 0.550 | 0.529 | 0.585 | 0.509 | 0.460 | 0.471 | 0.481 | 0.882 | 0.893 | 1.000 |

**Note**: The pairwise similarity coefficient calculated by SCoT markers. Lanes 1–20: genotypes of the 20 *Physalis* samples (1–20) in Table 1
